# Soil Contamination with Europium Induces Reduced Oxidative Damage in *Hordeum vulgare* Grown in a CO_2_-Enriched Environment

**DOI:** 10.3390/plants12173159

**Published:** 2023-09-02

**Authors:** Hanaa E. A. Amer, Hamada AbdElgawad, Mahmoud M. Y. Madany, Ahmed M. A. Khalil, Ahmed M. Saleh

**Affiliations:** 1 Botany and Microbiology Department, Faculty of Science, University of Cairo, Giza 12613, Egypt; helbadawy@sci.cu.edu.eg; 2 Integrated Molecular Plant Physiology Research, Department of Biology, University of Antwerp, B–2020 Antwerp, Belgium; hamada.abdelgawad@uantwerpen.be; 3 Department of Botany and microbiology, Faculty of Science, Beni-Suef University, Beni-Suef 62511, Egypt; 4 Biology Department, Faculty of Science, Taibah University, Al-Madinah Al-Munawarah 41411, Saudi Arabia; mmadany@taibahu.edu.sa; 5 Biology Department, Faculty of Science at Yanbu, Taibah University, King Khalid Rd., Al Amoedi, Yanbu El-Bahr 46423, Saudi Arabia; amsuliman@taibahu.edu.sa

**Keywords:** europium, elevated CO_2_, *Hordeum vulgare*, oxidative stress, photosynthesis, soil contamination

## Abstract

The extensive and uncontrolled utilization of rare earth elements, like europium (Eu), could lead to their accumulation in soils and biota. Herein, we investigated the impact of Eu on the growth, photosynthesis, and redox homeostasis in barley and how that could be affected by the future CO_2_ climate (eCO_2_). The plants were exposed to 1.09 mmol Eu^3+^/kg soil under either ambient CO_2_ (420 ppm, aCO_2_) or eCO_2_ (620 ppm). The soil application of Eu induced its accumulation in the plant shoots and caused significant reductions in biomass- and photosynthesis-related parameters, i.e., chlorophyll content, photochemical efficiency of PSII, Rubisco activity, and photosynthesis rate. Further, Eu induced oxidative stress as indicated by higher levels of H_2_O_2_ and lipid peroxidation products, and lower ASC/DHA and GSH/GSSG ratios. Interestingly, the co-application of eCO_2_ significantly reduced the accumulation of Eu in plant tissues. Elevated CO_2_ reduced the Eu-induced oxidative damage by supporting the antioxidant defense mechanisms, i.e., ROS-scavenging molecules (carotenoids, flavonoids, and polyphenols), enzymes (CAT and peroxidases), and ASC-GSH recycling enzymes (MDHAR and GR). Further, eCO_2_ improved the metal detoxification capacity by upregulating GST activity. Overall, these results provide the first comprehensive report for Eu-induced oxidative phytotoxicity and how this could be mitigated by eCO_2_.

## 1. Introduction

The lanthanide, europium (Eu), is one of the light rare earth elements (LREEs) that are characterized by a lower atomic mass than ca 153 and a larger effective radius than 95 pm [1]. LREEs have many uses in industry and modern technology, such as flints for lighters, carbon arc lighting, additives in steel, surface polishing, rechargeable batteries in mobile phones and computers, and even automotive catalysts [2,3]. Moreover, owing to their stimulatory effects on nutrients uptake and/or chlorophyll biosynthesis, some REEs have been integrated in trace concentrations to plant fertilizers [4]. Inevitably, the extensive and uncontrolled application of REE-containing fertilizers could lead to their accumulation in soils and biota causing human health hazards via its transfer through the food chain [4,5]. In this context, the toxicity of REEs on bacteria [6] and plants [7,8] and its human health hazards have been assessed [9,10]. Regarding its phytotoxicity, REEs have been reported to dislodge essential metal ions from enzymes, deteriorate sulfhydryl proteins, and induce the production of reactive oxygen species (ROS) [11,12]. 

In fact, the current concentration range of Eu in the natural soils (0.1–2.2 mg/kg soil) is firmly small, however, due to its increasing utilization in industrial activities, the sharp elevation of Eu levels is likely [13]. In this regard, the phytotoxicity of Eu, at concentration ≥0.2 mmol, has been suggested [7,14]. Physiologically, because of the similarities between Eu^3+^ and Ca^2+^ in radius and chemical properties, Eu^3+^ could replace Ca^2+^ in calcium-modulated protein (CaM–Ca^2+^), thus affecting the calcium signal transduction pathways and the related physiological processes [14]. In a recent study, *Brassica napus* cells exposed to 200 μM Eu^3+^ showed a marked decrease in cell viability compared to the control [7]. To overcome this undesired impact, plants produce metal-binding metabolites, store metal chelates in vacuoles, or secrete them through the root system, and synthesize protective polymers like lignin and callose [12,15]. The detailed impact of Eu on plant oxidative status has not been elucidated; however, as a general response to oxidative-stress-inducing agents, plants activate molecular and enzymatic antioxidant defense systems to modulate the redox homeostasis [16,17]. The non-enzymatic molecules include carotenoids, ascorbic acid (ASC), glutathione (GSH), and polyphenols [12]. The enzymatic antioxidants include superoxide dismutase (SOD), catalase (CAT), peroxidase (POX), glutathione peroxidase (GPX), ascorbate peroxidase (APX), and the glutathione-ascorbate cycling enzymes, i.e., glutathione reductase (GR), dehydroascorbate reductase (DHAR), and monodehydroascorbate oxidoreductase (MDHAR). SOD convertsO_2_^−^ to O2 and H_2_O_2_; the latter is scavenged by POX, CAT, APX, and GPX. These enzymes prevent the possible biological reactions between H_2_O_2_ and macromolecules present in the plant cell, hence preventing any possible modification in their structure and function [18,19]. 

According to the National Oceanic and Atmospheric Administration (NOAA), the current global concentration of CO_2_ in the atmosphere (aCO_2_) is about 420 ppm [20]. CO_2_ elevation comes from burning fuels for transportation and power generation, manufactures, burning agricultural wastes, and many other practices. According to IPCC-SRES B2-scenario, by the year 2100, the level of CO_2_ may reach 620 ppm [21]. Although extreme elevation in the atmospheric CO_2_ levels represents a challenge for plants [22], the biofertilization effect of CO_2_ concentrations up to 620 ppm was reported by increasing the photosynthesis rate and, thus, enhancing the production of building blocks and energy needed for plant metabolism and life [23,24]. In addition, the possible role of elevated CO_2_ (eCO_2_) in mitigating the negative effects of various stressors on plants has been previously proved [25,26]. Interestingly, eCO_2_ has been suggested as an effective approach to deal with the contamination of cultivation matrices by heavy metals [16,17,27,28,29]. Elevated CO_2_ has been reported to reduce the phytotoxicity of HM through enhancing Rubisco and PEPC activities and modulating the redox homeostasis, on both ROS production (photorespiration and NADPH oxidase activity) and scavenging (enzymatic and non-enzymatic) levels [16,17,27,28,29]. So far, no literature has discussed the ameliorating action of eCO_2_ on the phytotoxicity imposed by REEs. 

Besides wheat, rice, and maize, barley (*Hordeum vulgare* L.) is the fourth most domesticated crop in world agriculture. Barley grains contain moderate amounts of protein and relative percentage of phosphorus, calcium, and small amounts of vitamins [30]. It is widely used in breads, soups, stews, and other healthy foods due to its high carbohydrate content [31]. Barley is also an important feed for ruminants. Additionally, barley fiber is suitable for factory farming [32]. According to the Food and Agriculture Organization (FAO), the global average of barley seed production is about 3.50 tons per hectare, with an estimated average harvested area of 504,000 hectare worldwide [33]. Unfortunately, the production of barley and other crops is threatened by metal toxicity which leads to oxidative damage, inhibition in the rate of photosynthesis and other crucial metabolic processes, disturbances in water uptake, mineral nutrition and hormonal balance, and, consequently, significant reductions in growth and yield [34]. Although the hazardous impact of heavy metals on barley has been documented, the impact of LREEs, e.g., Eu, has not been investigated. 

This investigation was conducted to analyze the oxidative damage imposed by a phytotoxic dose of Eu^+3^ (1.09 mmol Eu^3+^/kg soil) in barley and how that could affect C assimilation and plant growth and physiology. Further, due to its well-known positive impact on C assimilation in C3 plants, like barley, we have assessed the mitigating action of eCO_2_ (620 ppm) on Eu^3+^-stressed plants. The sole or combined effects of Eu^3+^ and eCO_2_ on the growth parameters, photosynthetic efficiency, accumulation of Eu in plant tissues, and levels of oxidative stress markers were assessed. Detailed changes in enzymatic and molecular ROS scavenging systems as well as the metabolites of ascorbate-glutathione cycle were investigated.

## 2. Results

### 2.1. Biomass Production

**Figure 1:**
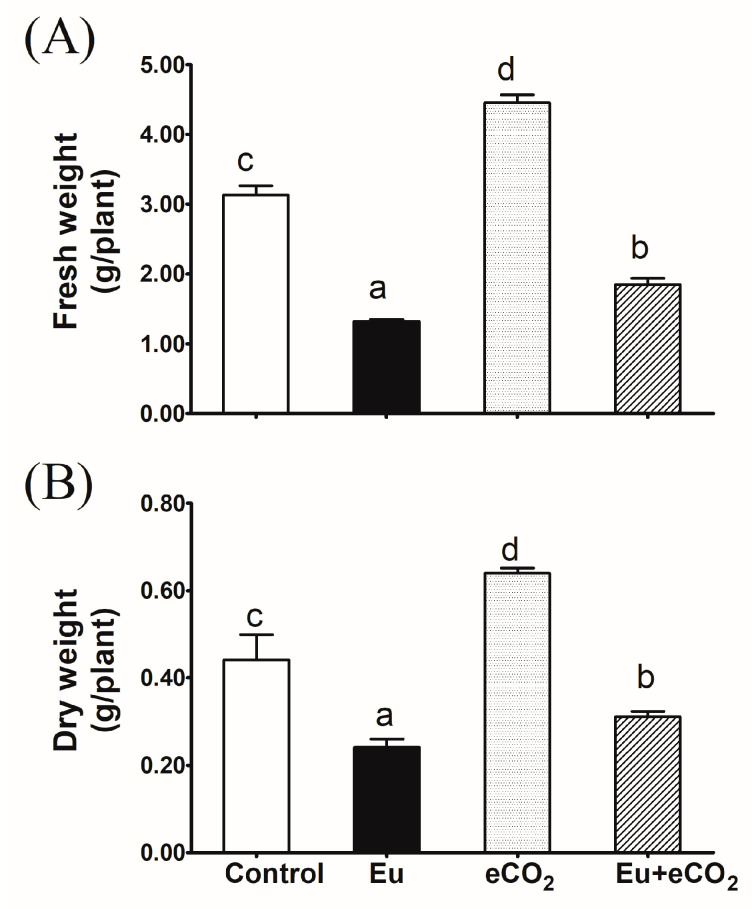
Effect of Eu, elevated CO_2_ (eCO_2_), and their combination (Eu + eCO_2_) on the fresh (**A**) and dry (**B**) biomasses of 28-day old *H. vulgare* plants. Each value represents the mean of five independent replicates and the vertical bars represent the standard error. Different lower-case letters on the bars, within the same graph, indicate significant difference at the 0.05 probability level as indicated by Tukey’s multiple range tests.

The estimated fresh and dry weights of 28-day old *H. vulgare* plants grown under control conditions were 3.1 and 0.44 g/plant, respectively (Figure 1A,B). The application of Eu dramatically decreased these values by 58 and 50%, respectively. Conversely, eCO_2_ spectacularly stimulated the plant biomass. The combination of Eu and eCO_2_ improved the biomass of the plants, compared to treatment with Eu alone, even though the values were significantly lower than the control.

### 2.2. Photosynthesis-Related Parameters

**Figure 2:**
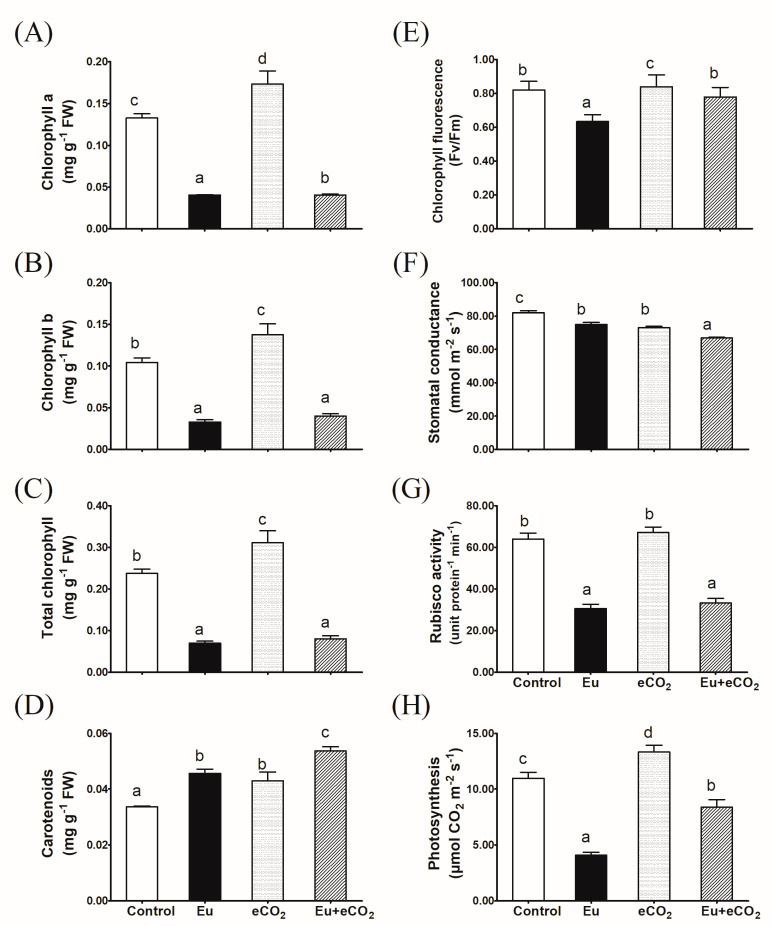
Effect of Eu, elevated CO_2_ (eCO_2_), and their combination (Eu + eCO_2_) on the photosynthesis-related parameters of 28-day old *H. vulgare* plants. (**A**): chlorophyll *a*; (**B**): chlorophyll *b*; (**C**): chlorophyll *a* + *b*; (**D**): carotenoids; (**E**): chlorophyll fluorescence; (**F**): stomatal conductance; (**G**): Rubisco activity; (**H**): rate of photosynthesis. Each value represents the mean of five independent replicates and the vertical bars represent the standard error. Different lower-case letters on the bars, within the same graph, indicate significant difference at the 0.05 probability level as indicated by Tukey’s multiple range tests.

The lowest value of chlorophylls *a* and *b* and their totals were recorded in plants treated with Eu (Figure 2A–C). The presence of eCO_2_ could not decrease the negative effect of Eu on chlorophylls, except for chlorophyll *a*. Eu, eCO_2_, and their combination significantly increased the carotenoid content in *H. vulgare* plants as compared to the control; however, a more pronounced improvement was caused by the combined treatment (Figure 2D). A significant decline in chlorophyll fluorescence was recorded through Eu application in the aCO_2_ environment (Figure 2E). However, the co-application of eCO_2_ treatment eliminated the negative impact of Eu on chlorophyll fluorescence. There were significant decreases in the values of stomal conductance due to the cultivation in the presence of Eu or eCO_2_ and to a greater extent under their synchronous application (Figure 2F). Regardless of CO_2_ level, a dramatic and significant inhibition in Rubisco-specific activity was observed in Eu-treated plants (Figure 2G). eCO_2_ alone had no effect on Rubisco-specific activity. Figure 2H shows the inhibitory effect of Eu on the photosynthesis rate of *H. vulgare* plants, as it dropped to half of the control value. On the contrary, eCO_2_ alone significantly improved the rate of photosynthesis. Further, a curative effect of eCO_2_ was recorded in Eu-treated plants. 

### 2.3. Stress Markers, Antioxidant Molecules, and GST Activity

Eu content, the levels of stress markers (H_2_O_2_ and MDA), antioxidant molecules (total polyphenols and flavonoids), the total antioxidant capacity (FRAP), and GST activity are presented in Table 1. Tracking the amount of Eu inside the shoots of barley plants treated with Eu under aCO_2_ revealed about 39 mg Eu g^−1^ DW; this value dropped to 44% by introducing 620 ppm eCO_2_. Eu-treated *H. vulgare* plants accumulated about 70% more H_2_O_2_ and MDA than the control. These elevations were significantly decreased through the co-application of eCO_2_. The lowest content of flavonoids was recorded in the Eu application, and the highest was observed under the presence of eCO_2_ alone. The contents of polyphenols increased in all treatments, where the highest value was detected in plants co-treated with Eu and eCO_2_. A similar trend was observed for FRAP. GST content only exceeded the control value in plants treated with Eu in an eCO_2_ atmosphere. 

### 2.4. Antioxidant Enzyme Activity

**Figure 3:**
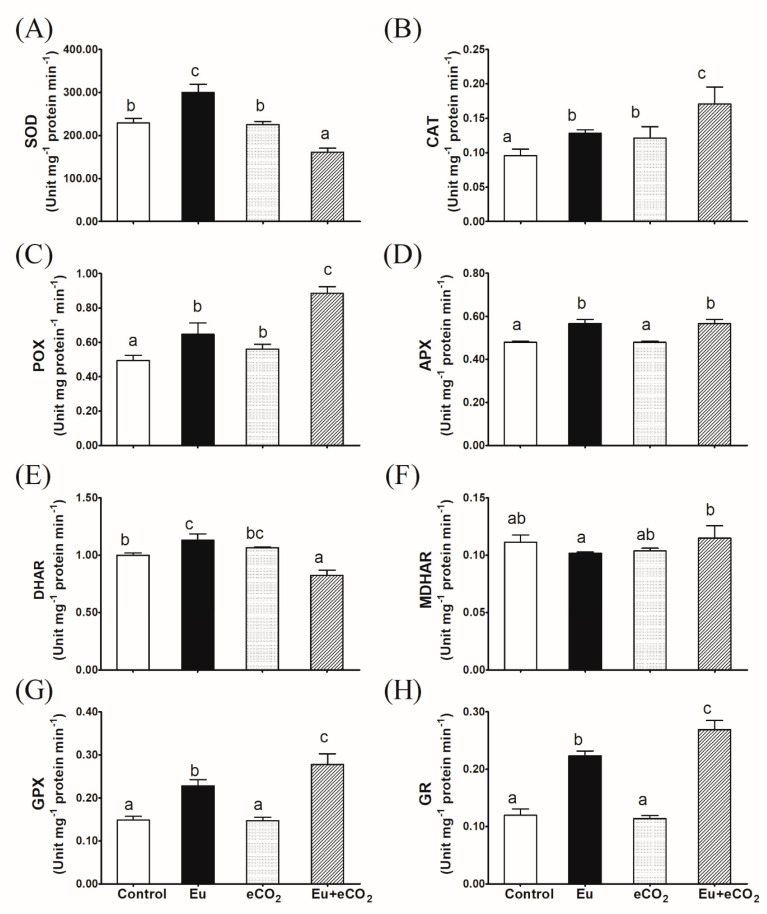
Effect of Eu, elevated CO_2_ (eCO_2_), and their combination (Eu + eCO_2_) on the activity of antioxidant enzymes in the shoots of 28-day old *H. vulgare* plants. (**A**): SOD, superoxide dismutase; (**B**): CAT, catalase, (**C**): POX, peroxidase; (**D**): APX, ascorbate peroxidase, (**E**): DHAR, dehydroascorbate reductase; (**F**): MDHAR, monodehydroascorbate reductase; (**G**): GPX, glutathione peroxidase; (**H**): GR, glutathione reductase. Each value represents the mean of five independent replicates and the vertical bars represent the standard error. Different lower-case letters on the bars, within the same graph, indicate significant difference at the 0.05 probability level as indicated by Tukey’s multiple range tests.

The activity of SOD significantly improved in Eu-treated plants but declined in plants treated with Eu + eCO_2_ (Figure 3A). A stimulatory effect of both Eu and eCO_2_ was observed in CAT and POX activities, which was more obvious with the synchronous application of Eu and eCO_2_ (Figure 3B,C). The activity of ascorbate-metabolizing enzymes, APX, DHAR, and MDHAR are presented in Figure 3D–F. APX was significantly stimulated by Eu under either aCO_2_ or eCO_2_; however, it was not affected by the application of eCO_2_ alone. DHAR activity significantly improved with Eu alone treatment, but the co-application of eCO_2_ caused a counteracting effect. For MDHAR activity, insignificant changes were recorded in all treatments. Regarding the enzymes that used glutathione as a substrate, GPX and GR, the activities of the two enzymes were enhanced by Eu and, to a greater extent, by Eu + eCO_2_ treatments (Figure 3G,H). 

### 2.5. ASC—GSH Cycle Metabolites 

**Figure 4:**
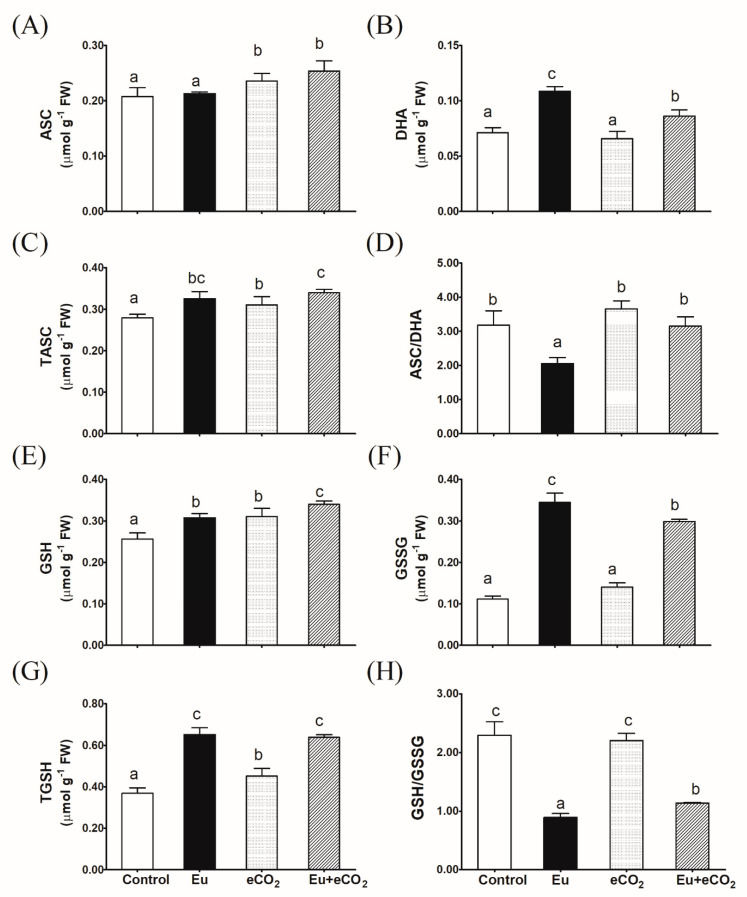
Effect of Eu, elevated CO_2_ (eCO_2_), and their combination (Eu + eCO_2_) on the levels of metabolites of glutathione-ascorbate cycle in the shoots of 28-day old *H. vulgare* plants. (**A**): ASC, reduced ascorbate; (**B**): DHA, oxidized ascorbate; (**C**): TASC, total ascorbate; (**D**): ASC/DHA ratio; (**E**): GSH, reduced glutathione; (**F**): GSSG, oxidized glutathione; (**G**): TGSH, total glutathione; (**H**): GSH/GSSG ratio. Each value represents the mean of five independent replicates and the vertical bars represent the standard error. Different lower-case letters on the bars, within the same graph, indicate significant difference at the 0.05 probability level as indicated by Tukey’s multiple range tests.

The Eu-alone treatment increased the level of DHA in *H. vulgare* plants by about 57%, compared to the control; however, it had no significant impact on the ASC level (Figure 4A,B). In contrast, eCO_2_ alone statistically stimulated the production of endogenous ASC but not DHA. The synchronous application of Eu + eCO_2_ recovered the negative impact of the Eu-alone treatment on the ASC/DHA ratio (Figure 4D). Eu or eCO_2_ alone and in combination significantly increased the content of GSH and TGSH (Figure 4E,G). However, the levels of GSSG were only improved in the presence of Eu, either under aCO_2_ or eCO_2_. In this regard, Eu caused a 3-fold increase in the level of GSSG in plants grown under aCO_2_ (Figure 4F). Expectedly, a dramatic drop in the GSH/GSSG ratio was observed in the Eu-alone treatment (Figure 4H). Such an Eu-induced reduction in the GSH/GSSG ratio was attenuated under an eCO_2_ atmosphere. 

## 3. Discussion

### 3.1. The Accumulation of Eu in Plant Shoots Slows Growth and Photosynthesis

So far, the impact of Eu on plant growth and physiology is rarely investigated. Thus, a comprehensive investigation of how Eu accumulation in soils could affect plant fitness, under current and future climatic regimes, is worthwhile. Herein, we have investigated the effects imposed by soil contamination with Eu (1.09 mmol Eu^3+^/kg soil) on biomass production, the crucial physiological process, photosynthesis, and redox homeostasis in barley plants grown under ambient (420 ppm) and elevated (620 ppm) levels of CO_2_. The significant accumulation of Eu in shoot tissues affirms its uptake and translocation (Table 1). In fact, there is evidence supporting the direct entrance of REEs into the plant cells [35]. In this regard, by measuring its fluorescence intensity, Eu was reported to be accumulated in the protoplast, plasma membrane, mitochondria, and cytoplasm [36,37]. The leaves of winter rye seem a little browner because of europium uptake and transport into the leaves [38]. Within the plant cell, Eu could alter the key plant physiological processes by interfering with enzyme activities or signaling pathways. For instance, due to the similarity between Eu^3+^ and Ca^2+^, in radius and chemical properties, Eu^3+^ was reported to replace Ca^2+^ in calcium-modulated proteins (CaM–Ca^2+^), thus affecting the calcium signal transduction pathways and the related physiological processes [14]. In accordance, the present results revealed a significant decline in plant biomass production (Figure 1), which was consistent with the inhibition in the photosynthesis-related parameters, i.e., chlorophyll content, the photochemical efficiency of PSII (Fv/Fm), and Rubisco activity (Figure 2). The reduction in plant biomasses, as affected by metal toxicity, has been regarded as a logical result for the deleterious effects on crucial processes like C fixation and nutrient utilization [39]. In this context, *Brassica napus* cells exposed to 200 μM Eu^3+^ showed a marked decrease in cell viability compared to the control [7]. Eu_2_O_3_ nanoparticles have reduced the least growth of and chlorophyll content in algal culture [40]. Eu may displace essential elements like Mn, K, and Mg, therefore causing disturbances in PS-II and impairing the photosynthetic electron transport chain [17]. It is worth noting that the impact of Eu on plant cells is a function of the applied dose where the lower dose might be promotive. For instance, an increase in *Daucus carota* cell vitality could be seen for 30 μM Eu (III), particularly at the beginning of the exposure [8]. This increase in cell vitalities could be attributed to the increased metabolic activities in response to the stress conditions [41].

### 3.2. Elevated CO_2_ Reduces the Accumulation of Eu and Mitigates its Impact on Growth and Photosynthesis

Interestingly, the synchronous application of eCO_2_ with Eu reduced the accumulation of Eu in plant shoots by about 40%. This reduction could be ascribed to the reduced stomatal conductance and the induced biosynthesis of polyphenols. In this regard, the reduction in stomatal conductance has been reported in plants treated with eCO_2_, especially under stress conditions [16,17]. Further, it has been reported that polyphenols, through their exudation to the soil, could reduce the bioavailability of heavy metals by acting as metal chelators [42]. The positive impact of eCO_2_ on the biosynthesis of polyphenols was reported in several plants [23,24]. Increased soil retention was in match with higher levels of phenolics in wheat and soybean plants [28]. In accordance, AbdElgawad et al. [16] recorded a reduced accumulation of Cr in two rice cultivars under CO_2_-enriched conditions. However, Saleh et al. [29] and Saleh et al. [17] reported that eCO_2_ had no significant impact on the uptake of Ni and Hg ions in wheat and maize, respectively. Thus, the impact of eCO_2_ on metal accumulation depends on the plant species and the tested metal. 

Elevated CO_2_ has been shown to exhibit fabulous effectiveness in alleviating the harmful effects of HM like Cd, Hg, Cu, and As on growth and physiology, especially in C3 plant systems [29,43,44,45]. As a substrate for Rubisco, eCO_2_ could improve photosynthesis, leading to the accumulation of the non-structural carbohydrates that afford the precursors and provide the energy for growth and development [29,46]. Further, the eCO_2_-induced carbohydrate accumulation could indirectly affect plant growth and physiology via upregulating biosynthesis of plant hormones like auxins [47]. In accordance, the co-application of 620 ppm eCO_2_ antagonized the negative impact of Eu on the measured photosynthesis-related parameters (Rubisco, chlorophyll fluorescence, and net photosynthetic rate), and thereby improved the growth of barley plants (Figure 1 and Figure 2). To date, there are no previous studies on the synchronous effects of Eu and eCO_2_ on plants, but several reports have shown the mitigating action of eCO_2_ on the phytotoxicity of heavy metals. For instance, our previous study confirmed that the synchronous application of eCO_2_ with mercuric oxide nanoparticles markedly mitigated their deleterious effects on activities of Rubisco and phosphoenol-pyruvate carboxylase and the rate of photosynthesis, and hence, enhanced maize growth [17]. Further, eCO_2_ mitigated the phytotoxicity of As and Cr on the growth and photosynthesis of wheat, soybean, and rice [16,27,28].

### 3.3. Eu Disrupts the Redox Homeostasis in Barley Plants but eCO_2_ Has an Antagonizing Action

Besides the negative impact of Eu on biomass and photosynthesis, Eu treatment increased the generation of ROS in barley plants, as indicated by higher levels of H_2_O_2_, the principle ROS generated in stressed plants [48]. Like most HMs, Eu causes stomatal closure, leading to insufficient intracellular CO_2_ concentration, which leads to the formation of singlet oxygen that severely damages PSI and PSII, and therefore disrupts the photosynthetic dynamicity [49]. Further, the reduced concentration of intracellular CO_2_ favors photorespiration (the oxygenation reaction of Rubisco), which is considered as a principal mechanism for H_2_O_2_ production [50]. The overproduction of ROS induces the accumulation of molecular antioxidants and the activities of antioxidant enzymes, which represents a confirmed strategy in plants to face different stresses [39,51]. Herein, plants treated with Eu, under an aCO_2_ climate, induced the ROS-scavenging molecules (polyphenols and carotenoids) and enzymes (CAT and peroxidases) as well as the ASC-GSH recycling enzymes (DHAR and GR). However, unfortunately, such levels of improvements were not enough to modulate the redox homeostasis and overcome the oxidative damage caused by Eu, as indicated by higher levels of MDA and disturbance in ASC/DHA and GSH/GSSG redox balances. 

Interestingly, growing plants treated with Eu in an eCO_2_ environment supported the non-enzymatic (carotenoids, flavonoids, and polyphenols) and enzymatic (CAT, POX, and GPX) antioxidants. In addition, eCO_2_ maintained the ASC/DHA and GSH/GSSG redox homeostasis as indicated by further improvements in the activities of MDHAR and GR. Such corroborative action of eCO_2_ on antioxidant defense mechanisms was evident in lower levels of H_2_O_2_ and MDA. In accordance, the levels of H_2_O_2_, MDA, and protein carbonyls, the product of protein oxidation, were highly reduced in response to eCO_2_ in Hg-stressed rice plants [17]. They ascribed such a decrease in the oxidative stress markers to the supporting action of eCO_2_ on molecular and enzymatic antioxidants as well as the recycling of ASC and GSH. More or less similar results were recorded in metal-stressed plants (either as bulk or nano forms) grown in CO_2_-enriched atmospheres [16,17,28,29,52,53]. Another suggested mechanism whereby eCO_2_ mitigated the oxidative stress induced by Eu is modulating ROS at the production level through the inhibition of photorespiration [50]. This hypothesis is supported by the earlier studies that recorded the marked inhibition in the activities of photorespiration enzymes (glycolate oxidase and hydroxymethylbutenyl diphosphate reductase) and the reduction in the glycine/serine ratio, as a measure for photorespiration rate, in heavy-metal-stressed C3 plants when exposed to eCO_2_ [16,28,44]. Moreover, we noticed a significant increase in the activity of GST through the co-application of Eu and eCO_2_, which supports the detoxification of Eu, due to the well-known role of GST in the detoxification of heavy metals and xenobiotics [54]. 

## 4. Materials and Methods

### 4.1. Plant Growth and Treatments

A homogenous quantity of surface-sterilized (sodium hypochlorite, 5% *v*/*v*) barley grains (*Hordeum vulgare* L.) was seeded in 15 cm depth × 13 cm diameter pots occupied by an artificial soil (30% Tref EGO substrates, Moerdijk, The Netherlands, mixed with 70% sterilized sand). Ten seeds were sown in each pot then thinned to 5 after 7 days from emergence. The soil composition for 1 g air-dried soil was as follows: carbon (11.7 mg), nitrate-nitrogen (14.8 mg), ammonium-N (1.1 mg), phosphorus (9.4 mg), and the humidity was 0.33 g water. Plants were arranged in 4 set-ups: (1) ambient CO_2_ (aCO_2_, 420 ppm CO_2_ (control)); (2) 1.09 mmol Eu^3+^/kg soil under aCO_2_ + (Eu); (3) elevated CO_2_ (eCO_2_, 620 ppm); and (4) 1.09 mmol Eu^3+^/kg soil under eCO_2_ + (Eu + eCO_2_). Eu was applied as EuCl_3_·6H_2_O (Sigma-Aldrich, Munich, Germany). To obtain the desired concentration of Eu^3+^, the soil was uniformly mixed with a definite amount of EuCl_3_·6H_2_O stock solution and then air-dried. The Eu^3+^ concentration was designated according to a preliminary experiment, based on growth retardation and the accumulation of oxidative stress markers, and the eCO_2_ level was identified based on the IPCC-SRES B2-state estimation of eCO_2_ of the year 2100 Murray and Ebi [21]. The pots were kept in a growth chamber at 21/18 °C in a 16/8h day/night photoperiod (150 μmol PAR m^−2^ s^−1^, 60% humidity). The pots were randomly organized in the growth chamber and watered daily to stabilize soil water content to 65%. To decrease bias, the experiment was duplicated by exchanging the two CO_2_ levels among the cabinets. Following four weeks after sowing, the plants were collected, the shoots’ and roots’ fresh and dry weights were identified, and the soil from rhizosphere was collected. Plant materials were taken in liquid N and kept at −80 °C. Soil samples were taken in an ice box and kept at −20 °C for further investigation. Treatments were completed with five replicates.

### 4.2. Photosynthesis-Related Parameters

The stomatal conductance (gs) and light-saturated photosynthetic rate (Asat) were evaluated from the fully enlarged youngest leaves (LI-COR LI-6400, LI-COR Inc., Lincoln, NE, USA) as reported by Abdelgawad et al. [55]. Photosynthetic pigment content was detected in acetone extract and estimated according to Porra et al. [56]. From the dark-adapted leaves of four-week-old plants, chlorophyll fluorescence was estimated via a fluorimeter (PAM2000, Walz, Effeltrich, Germany) over a 30 min period.

### 4.3. Determination of Eu Accumulation

For Eu extraction, the dried plant samples at 70 °C were processed in 13 M nitric acid at 185 °C for 25 min [57]. Afterward, element concentration was measured with a quadrupole inductively coupled plasma mass spectrometer (ICP-MS; model 820-MS) connected with glass nebulizer at 0.4 mL/min. The external standards were made at concentrations of 1–600 μg/L for calibration curves. As an internal standard during extraction, yttrium was additionally added to adjust nebulizer efficacy. Standard minerals were made in 0.23 M nitric acid. 

### 4.4. Oxidative Stress Markers 

As an indicator for lipid peroxidation, malondialdehyde (MDA) was detected in plant materials using the thiobarbituric acid method according to Hodges et al. [58]. For detecting hydrogen peroxide (H_2_O_2_), the xylenol orange approach was used following plant materials extraction in trichloroacetic acid (0.1%) as reported by Jiang et al. [59].

### 4.5. Total Antioxidant Capacity and Antioxidant Metabolites

Liquid-N2 was used for grinding plant materials that extracted in 80% ice-cold ethanol (2 mL) using a MagNALyser. The TAC of the extracts was determined with Ferric reducing/antioxidant power (FRAP) assay by a microplate reader at 600 nm [60]. Trolox was used as standard. Reduced glutathione (GSH) and reduced ascorbate (ASC) were determined with HPLC analysis [61]. After reduction with DTT, the total ascorbate (ASC + DHA) and glutathione (GSH + GSSG) content were evaluated. Ethanol (80%, *v*/*v*) was used for the extraction of phenolic compounds. Afterward, a spectrophotometer (Shimadzu UV-Vis 1601 PC, Japan) was used for polyphenol [62] and flavonoid [63] measurements. Following hexane extraction, extracts (CentriVap concentrator, Labconco, KA, USA) were taken for tocopherols detection using HPLC (Shimadzu, Hertogenbosch, The Netherlands; normal phase conditions, Particil Pac 5 µm column material, length 250 mm, i.d. 4.6 mm). In total, 5 ppm of dimethyl tocol was applied as an internal standard. 

### 4.6. Antioxidant Enzymes and Glutathione-S-Transferase

MagNALyser (Roche, Vilvoorde, Belgium) was used for antioxidant enzyme extraction in potassium phosphate buffer (50 mM, pH 7.0) accompanied with polyvinyl pyrrolidone (10%, *w*/*v*), Triton X-100 (0.25%, *v*/*v*), phenylmethylsulfonyl fluoride (1 mM), and ASC (1 mM). Subsequently, samples were centrifuged for 10 min (13,000 rpm, 4 °C) and the supernatant was taken for the activity evaluation of catalase (CAT, EC1.11.1.6), superoxide dismutase (SOD, EC 1.15.1.1), ascorbate peroxidase (APX, EC 1.11.1.11), peroxidase (POX, EC 1.11.1), glutathione reductase (GR, EC1.6.4.2), glutathione peroxidase (GPX, EC 1.11.1.9), monodehydroascorbate reductase (MDHAR, EC 1.6.5.4), and dehydroascorbate reductase (DHAR, EC 1.8.5.1). 

The activity of SOD was evaluated from the inhibition of nitroblue tetrazolium reduction at 560 nm [64]. The activity of POX was detected via the evaluation of the pyrogallol oxidation (ε430 = 2.47 mM^−1^·cm^−1^) following the method approved by Kumar and Khan [65]. The activity of CAT was examined by assessing the dissociation of hydrogen peroxide at 240 nm (ε240 = 0.0436 mM^−1^·cm^−1^) according to Aebi [66]. APX, MDHAR, DHAR, and GR activities were measured as described by Murshed et al. [67]. The activity of GPX was analyzed by quantifying NADPH oxidation decrement at 340 nm (ε340 = 6.22 mM^−1^·cm^−1^) as reported by [48]. Potassium phosphate buffer (50 mM, pH 7.0) was used for the extraction of glutathione-S-transferase (GST, EC 2.5.1.18) and further measured [68]. The soluble proteins in the extracts were determined according to the method reported by Lowry et al. [69].

### 4.7. Statistical Analyses

Experiments were conducted in a completely randomized block design. Data analyses were performed using the Statistical Analysis System (SPSS Inc., Chicago, IL, USA). Data normality and the homogeneity of variances were tested using the Kolmogorov–Smirnov test and Levene’s test, respectively. All the data were treated with one-way analysis of variance (ANOVA). Tukey’s test (*p* ≤ 0.05) was conducted as the post hoc test for separation of means. Five replicates for each experiment were conducted (*n* = 5).

## 5. Conclusions

This study provides the first report regarding the interactive effect of Eu and eCO_2_ on the growth and physiology of plants. Besides presenting a comprehensive investigation for the oxidative phytotoxicity induced by Eu accumulation in soils, Eu, at a concentration of 1.09 mmol Eu^3+^/kg soil, caused sharp reductions in growth and photosynthesis and induced sever oxidative damage in the treated plants. Compared to Eu alone, the co-application of eCO_2_ improved plant biomass and photosynthesis efficiency and mitigated Eu-induced oxidative damage. Such positive implications of eCO_2_ could be ascribed to two main plausible strategies including: (1) the recovery of the deleterious effect of Eu on the photochemical efficiency of PS II (Fv/Fm) and, hence, mitigating the photosynthesis rate; and (2) modulating ROS homeostasis at both production and detoxification levels.

## Figures and Tables

**Table 1:** Effect of Eu, elevated CO_2_ (eCO_2_), and their combination (Eu + eCO_2_) on the accumulation of Eu and the contents of stress markers (H_2_O_2_ and MDA), antioxidant molecules (total polyphenols and flavonoids), total antioxidant capacity (FRAP), and glutathione-S-transferase (GST) activity in the shoots of 28-day old *H. vulgare* plants. Values are presented as the mean of five independent replicates ± standard error. Different lower-case letters in the same row indicate significant difference at the 0.05 probability level as indicated by Tukey’s multiple range tests.

Parameter	Control	Eu	eCO_2_	Eu + eCO_2_
Eu content (mg g^−1^ DW)	ND	38.69 ± 1.25 b	ND	21.65 ± 0.52 a
H_2_O_2_ (nmol g^−1^ FW)	562.49 ± 13.63 b	964.04 ± 45.26 d	495.1 ± 24.88 a	708.33 ± 13.6 c
MDA (nmol g^−1^ FW)	2.48 ± 0.06 a	4.31 ± 0.24 c	2.33 ± 0.23 a	3.61 ± 0.35 b
FRAP (mmol trolox g^−1^ FW)	36.77 ± 2.87 a	46.07 ± 1.54 b	43.55 ± 0.61 b	54.71 ± 2.28 c
Polyphenol (mg gallic acid g^−1^ FW)	15.23 ± 0.4 a	20.53 ± 1.07 c	18.94 ± 0.46 b	25.32 ± 1.26 d
Flavonoids (mg quercetin g^−1^ FW)	35.02 ± 1.37 c	26.07 ± 1.07 a	37.47 ± 1.34 d	29.27 ± 0.81 b
GST (unit mg^−1^ protein min^−1^)	0.19 ± 0.01 a	0.18 ± 0.01 a	0.19 ± 0 a	0.27 ± 0.01 b

ND means not detected.

## Data Availability

The data generated and analyzed during this study are included in this article.
